# The Prognostic Implication of the BRAF V600E Mutation in Papillary Thyroid Cancer in a Chinese Population

**DOI:** 10.1155/2022/6562149

**Published:** 2022-06-16

**Authors:** Ziheng Ye, Xiaotian Xia, Peipei Xu, Wenfei Liu, Shoufei Wang, Youben Fan, Minggao Guo

**Affiliations:** Center of Thyroid and Parathyroid, Department of Thyroid, Parathyroid, Breast and Hernia Surgery, Shanghai Jiao Tong University Affiliated Sixth People's Hospital, 600 Yi-Shan Road, Shanghai 200233, China

## Abstract

**Background:**

The BRAF V600E mutation is an important genetic event in papillary thyroid cancer (PTC). This study aimed to provide additional information regarding the association of the BRAF V600E mutation with PTC prognosis.

**Methods:**

A retrospective single-center study based on a Chinese population was performed to analyze the association of the BRAF V600E mutation with several clinicopathological features. Kaplan–Meier survival curves and Cox proportional hazards regression analysis were applied to implement the survival analysis.

**Results:**

The BRAF V600E mutation was present in 1102 (87.7%) of the 1257 patients and was significantly associated with older age, conventional subtype, multifocality, advanced TNM stage, and a reduced prevalence of Hashimoto's thyroiditis. The Kaplan–Meier survival curves demonstrated that the difference between the BRAF V600E-positive and BRAF V600E-negative groups was significant with a log-rank *P*-value of 0.048. The Cox proportional hazards regression analysis adjusted HR was 3.731 (95% CI, 1.457 to 9.554). We further demonstrated that larger tumor size (>1 cm), extrathyroidal extension (ETE), and lateral lymph node metastasis (LNM) were associated with a higher probability of PTC recurrence in patients harboring the BRAF V600E mutation.

**Conclusions:**

The BRAF V600E mutation remains an independent risk factor for PTC recurrence and may be useful for clinical decisions when it combines with some pathological factors.

## 1. Introduction

Thyroid cancer is the most common endocrine malignancy and accounts for 2.1% of all malignancies [1]. The most common histological type of thyroid cancer is papillary thyroid cancer (PTC), accounting for 85% of all thyroid malignancies [2]. PTC consists of several histological variants, and conventional PTC (CPTC) and follicular variant PTC (FVPTC) account for the majority of PTCs [3].

Although PTC is generally indolent and curable, disease recurrence is common after initial treatment, and it can cause disease-specific mortality [4]. A major clinical controversy is how to reliably distinguish the patients who need more radical treatment to reduce the risk of disease recurrence and progression from the large number of PTC patients around the world with indolent disease. Risk stratification is essential for clinical decisions regarding thyroid cancer, based on factors such as sex, patient age, tumor size, lymph node metastasis (LNM), distant metastasis, extrathyroidal extension (ETE), and disease staging [5]. However, the current risk stratification of PTC has some limitations, which are amplified by the high annual incidence of PTC.

Recently, prognostic molecular markers have been applied to improve the risk stratification of PTC, especially the BRAF V600E mutation. This mutation results in a valine-to-glutamic acid change in codon 600 of the BRAF protein, which may activate the mitogen-activated protein kinase (MAPK) signaling pathway in human cancer [6]. It is worth noting that the BRAF V600E mutation has received a great deal of attention because it may be the main oncogenic driver of PTC; it is found in 45% of patients on average [7]. A study published in 2012 showed that the prevalence of the BRAF V600E mutation has significantly increased over the last 15 years [8]. Several studies have documented an association of the BRAF V600E mutation with aggressive clinicopathological characteristics of PTC, including LNM, ETE, and disease recurrence [9, 10]. Additionally, BRAF V600E is expected to become a clinical factor used for determining the extent of the surgery along with other conventional risk factors [11].

However, there are many differences regarding the BRAF V600E mutation in the Chinese population. The prevalence of the BRAF V600E mutation in the Chinese population is substantially higher than that in western populations and has been reported to be 83.7% and 75.4% in two studies based on Chinese patients [12, 13]. Then, some Chinese studies have demonstrated that the BRAF V600E mutation is not the predictive factor for some aggressive clinicopathological characteristics, especially LNM [12–14]. Given these data, we investigated the association of the BRAF V600E mutation with some clinicopathological characteristics and PTC recurrence in a large single-center study in China to determine its value for clinical practice.

## 2. Methods

### 2.1. Study Patients

A total of 1257 patients who were diagnosed with primary PTC at the Shanghai Jiao Tong University Affiliated Sixth People's Hospital between January 2016 and April 2019 were enrolled in the present study, and 1167 patients who had follow-up data were included in the survival analysis.

All patients were treated with suitable and radical dissection, including total or hemithyroidectomy and therapeutic or prophylactic neck dissection, depending on the clinical standard indications. Pathological diagnoses of PTC and histological subtypes were confirmed by two experienced pathologists based on the World Health Organization criteria, and detailed pathological information, such as tumor size, ETE, multifocality, Hashimoto's thyroiditis, and the presence of LNM, was documented.

Postoperative treatments were dictated by standard criteria and consisted of conventional thyroid-stimulating hormone suppression at appropriate levels with or without radioiodine-131 treatments. Local, regional, and distant recurrences were all considered PTC recurrence, which was diagnosed per standard histologic, cytologic, radiographic, or biochemical criteria [5, 15]. Follow-up time was defined as the time from the initial surgical treatment to the discovery of a PTC recurrence or to the most recent clinic follow-up for patients without disease recurrence.

### 2.2. Study Design

This retrospective study was approved by the ethics committee of the Shanghai Jiao Tong University Affiliated Sixth People's Hospital, and written informed consent was obtained from all patients. Clinical information such as age, sex, and TNM stage of the tumor was retrieved from medical records. The TNM stage was defined based on the seventh edition of the American Joint Committee on Cancer (AJCC) staging system.

The BRAF V600E mutational analyses were performed by professional pathologists after the surgeries. DNA was extracted from formalin-fixed, paraffin-embedded (FFPE) tissue blocks via the use of a commercial kit (FFPE DNA reagent, Amoy Diagnostics Co. Ltd., Xiamen, China). The mutational analyses were conducted using a China Food and Drug Administration (CFDA)-approved human BRAF V600E ARMS-PCR kit (Amoy Diagnostics Co., Ltd., Xiamen, China). BRAF mutation status did not affect treatment decisions for either operative or postoperative treatments.

We analyzed the relationship between the BRAF V600E mutation and clinicopathologic features of PTC and determined the significance of the BRAF V600E mutation alone or in combination with other known risk factors for predicting the recurrence of PTC.

### 2.3. Statistical Analyses

Patient age, tumor size, and follow-up time, which were not normally distributed in this study, were summarized using median and quartiles. Categorical data are presented as frequencies and percentages and were analyzed using the chi-square test or Fisher's exact test as appropriate. The Mann-Whitney *U* test for two independent samples in nonparametric statistics was used to compare the TNM stages. Kaplan–Meier survival curves and log-rank tests were used to analyze recurrence-free survival. The Cox proportional hazards regression analysis was performed to calculate hazard ratios (HRs) and 95% confidence intervals (CIs) for the comparison of disease recurrence by BRAF V600E status with adjustment for confounding factors. All statistical analyses were conducted using the Statistical Package for Social Sciences for Windows (SPSS) version 25.0 (IBM Corp, NY, USA). All *P* values were two-sided, and a *P* value of less than .05 was considered statistically significant.

## 3. Results

### 3.1. Demographic Information of the Patients

We enrolled a total of 1,257 patients (909 women and 348 men) diagnosed with PTC at our medical center, with a median age of 42 years (interquartile range [IQR], 32 to 53 years). Among the patients, 1200 (95.5%), 36 (2.9%), and 21 (1.7%) were classified as conventional, follicular, and other subtypes, respectively. The BRAF V600E mutation prevalence in our study was 87.7% and 90.1% for PTC and CPTC, which was much higher than that observed in western populations. The overall PTC recurrence rate was 7.5%, which is relatively lower than previously reported [16]. For all PTC patients, the median follow-up time was 21 months (IQR, 15 to 29 months) in the BRAF V600E-positive group and 21 months (IQR, 15 to 30 months) in the BRAF V600E-negative group, with no significant differences. The demographic information of the patients is summarized in Table 1.

### 3.2. Relationship of the BRAF V600E Mutation with the Clinicopathological Characteristics of PTC

As shown in Table 1, the BRAF V600E-positive patients were significantly older than the BRAF V600E-negative patients, with a median age of 43 years (IQR, 33–53 years) versus 37 years (IQR, 29–49 years). In the BRAF V600E -positive group, CPTC accounted for 98.1% of cases (1,081 of 1,102), and FVPTC only accounted for 1.0%, which was significantly different from that of the BRAF V600E -negative group. It was worth noting that the BRAF V600E mutation had a significant association with multifocality and a more advanced TNM stage. These results might indicate that the BRAF V600E mutation is associated with more aggressive clinical features. Consistent with this indication, the presence of Hashimoto's thyroiditis, which is considered to be a protective factor in PTC patients [17], was significantly associated with BRAF V600E-negative patients. No significant relationship was observed between BRAF V600E status and other clinicopathological characteristics such as sex, tumor size, central LNM or distant metastasis. Moreover, similar results were found when only CPTC was analyzed, except for lateral LNM, a difference that had statistical significance in the PTC group but not in the CPTC group.

### 3.3. Relationship between BRAF V600E Mutation and Recurrence of PTC

As we stated above, there were 1,167 patients included in the survival analysis after the exclusion of 90 patients due to a lack of follow-up data. The overall recurrence rates of PTC were 8.1% and 3.4% for the BRAF V600E-positive and BRAF V600E-negative groups, respectively. When the analysis was restricted to CPTC, the recurrence rates of the BRAF V600E-positive and BRAF V600E-negative groups were 8.2% and 2.7%, respectively, showing an overt difference between the two groups.

**Figure 1:**
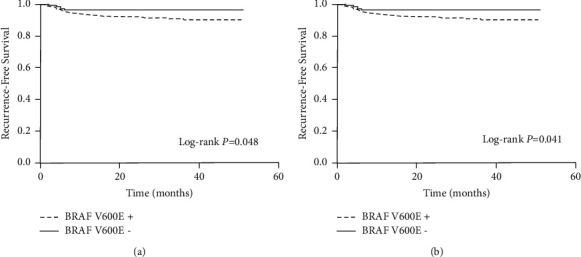
Kaplan–Meier survival analysis of disease recurrence-free survival of patients with PTC (a) and CPTC (b), stratified by BRAF V600E status.

The Kaplan–Meier survival analyses also demonstrated a significant association between the BRAF V600E mutation status and recurrence-free survival in the overall PTC group (Figure 1(a)) and the CPTC group (Figure 1(b)), with log-rank *P* values of 0.048 and 0.041, respectively. As listed in Table 2, with the comparison of the BRAF V600E status, the unadjusted HRs of the PTC group and the CPTC group were 2.409 (95% CI, 0.977 to 5.941) and 3.109 (95% CI, 0.982 to 9.842), respectively, with no significant difference. However, after adjustment for patient age, sex, tumor size, ETE, central LNM, lateral LNM, and distant metastasis, the adjusted HRs were 3.731 (95% CI, 1.457 to 9.554) and 3.993 (95% CI, 1.239 to 12.869) for the PTC and CPTC groups, respectively.

### 3.4. Several Pathological Factors Which Could Enhance the Risk of Recurrence in PTC Patients Harboring the BRAF V600E Mutation

**Figure 2:**
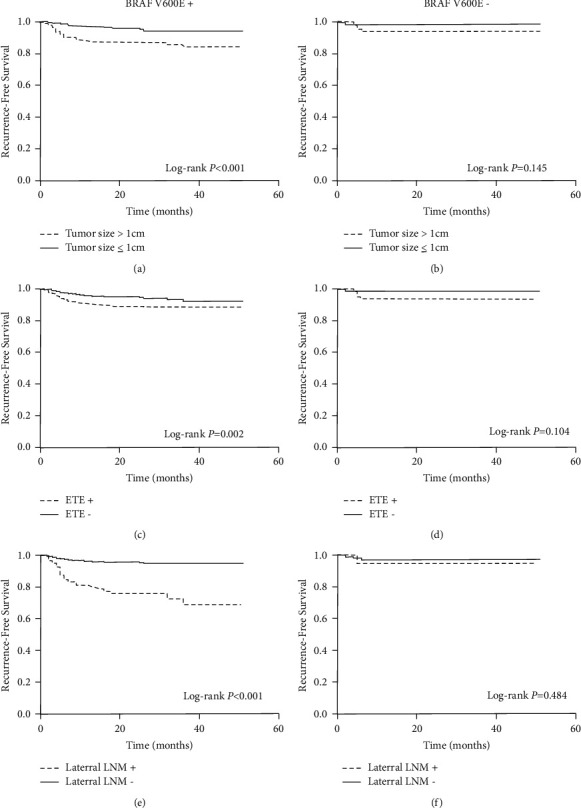
Kaplan–Meier survival analysis of disease recurrence-free survival of patients in the BRAF V600E-positive group (a, c, e) and the negative group (b, d, f), stratified by tumor size, ETE, and lateral LNM.

To identify the patients who had higher recurrence risks in the BRAF V600E-positive group, we further evaluated the influence of conventional pathological factors on PTC recurrence and finally found three pathological factors which were significant for the BRAF V600E-positive group exclusively. As shown in Figure 2(a), papillary thyroid microcarcinomas (PTMC) had better recurrence-free survival than tumors that were larger than 1 cm in the BRAF V600E-positive group (log-rank *P* value less than 0.001). Similarly, ETE and lateral LNM, which were considered to be more aggressive features in previous studies, had an enhanced risk of PTC recurrence in the BRAF V600E-positive group (Figures 2(c) and 2(e)). Conversely, these factors were not associated with recurrence-free survival in the BRAF V600E-negative group (Figures 2(b), 2(d), and 2(f)), with log-rank *P* values all greater than 0.05.

## 4. Discussion

Stratification of the prognosis of PTC patients is becoming a more urgent task because the prevalence of PTC has been increasing yearly. Recently, a number of promising molecular markers of PTC have emerged in clinical translational research, especially the BRAF V600E mutation [18].

The BRAF V600E mutation has drawn much attention as described in the Introduction section; however, it has also become embroiled in considerable controversy in several studies. Two large multicenter studies demonstrated an association between the BRAF V600E mutation and mortality and recurrence in patients with PTC based on western populations [10, 19], and both concluded that the BRAF V600E mutation was a significant molecular marker for the prognosis of PTC. In contrast, another study in the US showed that there was no significant relationship between the BRAF mutation and recurrence-free survival or disease-specific survival in long-term follow-up [20]. It is a pity that, currently, no research based on a Chinese population has provided sufficient evidence to illustrate the prognostic implication of the BRAF V600E mutation in PTC patients.

In our study, we found that the prevalence of the BRAF V600E mutation was 87.7% for all PTC patients and 90.1% for CPTC only, which is similar to the rates reported by other studies based on Chinese populations [12, 13]. Similarly, a study from Korea reported that BRAF V600E mutations were present in 83.7% of PTC patients (2789 of 3332) [21]. However, a meta-analysis that included 32 studies showed that the pooled prevalence of the BRAF V600E mutation was 50.9% [22], which is comparable to most studies based on western populations. Two reasons may contribute to the differences among these studies: The incidence of the BRAF V600E mutation is substantially higher in high-iodine regions [23], including China and Korea; the ARMS-PCR method we utilized is more sensitive at detecting BRAF V600E somatic mutations than DNA sequencing, which can detect as low as 1% mutant alleles [24].

We also investigated the relationship between the BRAF V600E mutation and clinicopathological characteristics and PTC recurrence. Although the BRAF V600E mutation was associated with some aggressive clinicopathological features, such as multifocality and advanced TNM stage, it failed to be associated with LNM. Moreover, we illustrated that the BRAF V600E mutation is an independent risk factor for PTC recurrence in Chinese populations, but it is improper to use the BRAF V600E mutation alone to influence clinical decisions because the high prevalence of the BRAF V600E mutation in China weakened its effectiveness for the prediction of PTC recurrence. Therefore, we further investigated the influence of several pathological factors on PTC recurrence that coexisted with the BRAF V600E mutation and suggested that the BRAF V600E mutation is still helpful for clinical treatment decisions when it is combined with some pathological factors.

To be specific, a more radical surgical procedure, such as total thyroidectomy and radioiodine-131 treatment, should be taken into account when the BRAF V600E mutation coexists with a large tumor size (>1 cm) or microscopic ETE in PTC patients. In addition, more radical postoperative management, including a higher dose of radioiodine-131 treatment and more frequent follow-up, should be implemented in relatively higher-risk patients who harbor the BRAF V600E mutation.

This study is subject to some limitations as follows. First, a selection bias might have occurred in our study, such as is often observed in single-center studies. A large consecutive cohort of patients with complete clinical data may be helpful to minimize this bias. Next, the follow-up time was relatively short in our study (median, 21 months; range, 13–51 months) in regards to the indolence of PTC. Nevertheless, most recurrence events should have been detected because PTC always recurs within the first several years after initial treatment [10]. Finally, radioiodine-131 treatment results were not recorded in our study because some of our patients received this treatment in various other medical centers, and we could not collect the relevant complete data. In addition, the patients with the BRAF V600E mutation might have received a higher dose of radioiodine because of their more advanced TNM stage, as described above. As a result, we might have underestimated the effect of the BRAF V600E mutation on PTC recurrence because radioiodine-131 treatment can reduce the risk of PTC recurrence [5].

A future research direction could be to investigate the interaction of the BRAF V600E mutation with other somatic genetic alterations in various signaling pathways, which could provide novel diagnostic and prognostic molecular markers and therapeutic targets for thyroid cancer [25].

## 5. Conclusion

In summary, we have reported the prevalence and clinicopathological associations of the BRAF V600E mutation and provided sufficient evidence to illustrate its relationship with PTC recurrence in a Chinese population. Furthermore, we suggest that the BRAF V600E mutation may be useful for clinical treatment decisions when it is combined with some pathological factors.

## Figures and Tables

**Table 1:** Demographic and clinicopathological characteristics of PTC and CPTC patients.

BRAF V600E status	PTC			CPTC		
	BRAF V600E-positive	BRAF V600E-negative	*P*	BRAF V600E-positive	BRAF V600E-negative	*P* value
Number of patients	1102 (87.7%)	155 (12.3%)		1081(90.1%)	119 (9.9%)	
Sex			0.088			0.162
Male	314 (28.5%)	34 (21.9%)		311 (28.8%)	27 (22.7%)	
Female	788 (71.5%)	121 (78.1%)		770 (71.2%)	92 (77.3%)	
Age (years)	43 [33–53]	37 [29–49]	<0.001	43 [33–53]	37 [29–49]	<0.001
≥45 y	517 (46.9%)	51 (32.9%)	0.001	503 (46.5%)	41 (34.5%)	0.012
Subtype			<0.001			—
CPTC	1081 (98.1%)	119 (76.8%)		—	—	
FVPTC	11 (1.0%)	25 (16.1%)		—	—	
Other	10 (0.9%)	11 (7.1%)		—	—	
Tumor size (cm)	1.0 [0.7–1.5]	1.0 [0.8–1.8]	0.174	1.0 [0.7–1.5]	1.0 [0.7–1.5]	0.649
>1 cm	466 (42.3%)	71 (45.8%)	0.407	458 (42.4%)	50 (42.0%)	0.941
Multifocality	396 (35.9%)	37 (23.9%)	0.003	390 (36.1%)	31 (26.1%)	0.030
ETE	568 (51.5%)	68 (43.9%)	0.074	556 (51.4%)	51 (42.9%)	0.076
Central LNM	556 (50.5%)	81 (52.3%)	0.674	550 (50.9%)	66 (55.5%)	0.342
Lateral LNM	185 (16.8%)	38 (24.5%)	0.018	181 (16.7%)	28 (23.5%)	0.064
Hashimoto's thyroiditis	281 (25.5%)	62 (40.0%)	<0.001	269 (24.9%)	49 (41.2%)	<0.001
Distant metastasis	15 (1.4%)	4 (2.6%)	0.279	15 (1.4%)	2 (1.7%)	0.682
TNM stage			0.011			0.020
I	717 (65.1%)	116 (74.8%)		706 (65.3%)	90 (75.6%)	
II	11 (1.0%)	5 (3.2%)		9 (0.8%)	3 (2.5%)	
III	281 (25.5%)	25 (16.1%)		275 (25.4%)	19 (16.0%)	
IV	93 (8.4%)	9 (5.8%)		91 (8.4%)	7 (5.9%)	
Follow-up time (months)^*∗*^	21 [15–29]	21 [15–30]	0.310	21 [15–29]	22.5 [17–32]	0.030

^*∗*^Data were from 1167 patients who had follow-up data.

**Table 2:** Recurrence and HRs for BRAF V600E-positive versus negative in PTC and CPTC patients.

	Recurrence					
	BRAF V600E-Positive	BRAF V600E-Negative	Hazard Ratio (HR)	*P*	Adjusted HR	*P* value
PTC	83 (8.1%)	5 (3.4%)	2.409 [0.977–5.941]	0.056	3.731 [1.457–9.554]	0.006
CPTC	82 (8.2%)	3 (2.7%)	3.109 [0.982–9.842]	0.054	3.993 [1.239–12.869]	0.020

## Data Availability

The data that support the findings of this study are available from the corresponding author upon reasonable request.
